# Detection and Validation of Circular DNA Fragments Using Nanopore Sequencing

**DOI:** 10.3389/fgene.2022.867018

**Published:** 2022-05-30

**Authors:** Alicia Isabell Tüns, Till Hartmann, Simon Magin, Rocío Chamorro González, Anton George Henssen, Sven Rahmann, Alexander Schramm, Johannes Köster

**Affiliations:** ^1^ Laboratory of Molecular Oncology, West German Cancer Center, Department of Medical Oncology, University Hospital Essen, Essen, Germany; ^2^ Algorithms for Reproducible Bioinformatics, Institute of Human Genetics, University Hospital Essen, University of Duisburg-Essen, Essen, Germany; ^3^ Institute for Artificial Intelligence in Medicine, IKIM, University Hospital Essen, Essen, Germany; ^4^ Department of Pediatric Oncology/Hematology, Charité-Universitätsmedizin Berlin, Berlin, Germany; ^5^ Max-Delbrück-Centrum für Molekulare Medizin (BIMSB/BIH), Berlin, Germany; ^6^ Berlin Institute of Health, Berlin, Germany; ^7^ German Cancer Consortium (DKTK), Partner Site Berlin and German Cancer Research Center (DKFZ), Heidelberg, Germany; ^8^ Experimental and Clinical Research Center (ECRC) of the MDC and Charité Berlin, Essen, Germany; ^9^ Center for Bioinformatics and Department of Computer Science, Saarland University, Saarbrücken, Germany

**Keywords:** cancer, circular DNA, nanopore sequencing, algorithm, snakemake

## Abstract

Occurrence of extra-chromosomal circular DNA is a phenomenon frequently observed in tumor cells, and the presence of such DNA has been recognized as a marker of adverse outcome across cancer types. We here describe a computational workflow for identification of DNA circles from long-read sequencing data. The workflow is implemented based on the Snakemake workflow management system. Its key step uses a graph-theoretic approach to identify putative circular fragments validated on simulated reads. We then demonstrate robustness of our approach using nanopore sequencing of selectively enriched circular DNA by highly sensitive and specific recovery of plasmids and the mitochondrial genome, which is the only circular DNA in normal human cells. Finally, we show that the workflow facilitates detection of larger circular DNA fragments containing extrachromosomal copies of the MYCN oncogene and the respective breakpoints, which is a potentially useful application in disease monitoring of several cancer types.

## 1 Introduction

In the nucleus of eukaryotic cells, DNA is almost exclusively found in linear chromosomes, which are hierarchically organized. Their basic unit is the nucleosome, which consists of 146 bp of double-stranded DNA wrapped around a histone octamer. Packing of nucleosomes forms a helical structure known as the 30 nm chromatin fiber, which coils into highly condensed chromosomes during metaphase in cell division (Annunziato, 2008). In addition to intact chromosomes, Cox et al. (1965) observed very small double chromatin bodies during chromosome level analyses of human tumors. Although the concept of extrachromosomal DNA is therefore not new, its role in cancer biology and progression has only been investigated more thoroughly in recent years due to technological advances in sequencing methods and high-resolution microscopy.

Since then, various types of extrachromosomal circular DNAs (eccDNA) including telomeric circles (t-circles, occurring in cells with alternative lengthening of telomeres [ALT]), small polydispersed DNA elements (100 bp–10 kbp), microDNAs (100–400 bp), and extrachromosomal DNA (ecDNA) (one to three Mbp) have been identified (Cohen et al., 1997; Henson et al., 2009; Fan et al., 2011; Shibata et al., 2012). The majority of eccDNAs found in healthy cells is smaller than 1 kb and lacks coding regions. However, large ecDNAs are rarely found in healthy cells but are present in 46% of cancer cell lines (Turner et al., 2017). EcDNAs are characterized by large circles containing entire genes or regulatory elements while lacking centromeres and telomeres. They can be observed using light microscopy (Verhaak et al., 2019). Several studies have shown that presence of ecDNAs in cancer cells correlates with high oncogene copy numbers, high intratumoral heterogeneity and poor patient outcome (Turner et al., 2017; Kim et al., 2020).

EcDNAs replicate approximately once per cell cycle (Ruiz et al., 1989) but are not segregated equally between mother and daughter cells due to the aforementioned lack of centromeres. This enables cells to evolve from a homogeneous population to a heterogeneous pool with respect to copy numbers of ecDNA. In addition to elevated copy numbers, ecDNAs are less compact compared to normal chromatin, rendering genes and regulatory elements more accessible for the transcriptional machinery. This in turn could explain why oncogenes encoded on ecDNAs are among the most highly expressed genes of the tumor transcriptome (Wu et al., 2019). Further, extrachromosomal DNAs promote genome remodelling through chimeric circularization and reintegration into the linear genome, thereby disrupting tumor suppressor genes or enhancing oncogene expression (Koche et al., 2020). EcDNAs thus increase intratumoral heterogeneity, which provides a growth advantage by enabling tumor cells to evolve rapidly under selective pressure. Overcoming the technical challenges to reliably detect ecDNAs would thus pave the way to define ecDNAs as promising new targets for tumor diagnostics and therapy.

Since extrachromosomal DNAs are large and can contain sequences from multiple genomic sites, their full length reconstruction using short read sequencing approaches may cause errors in read mapping and *de-novo* assembly (Treangen and Salzberg, 2012). Long read sequencing approaches such as nanopore sequencing can help to overcome these problems, as single reads can already cover multiple breakpoints and may increase robustness of ecDNA detection. Additionally, nanopore sequencing technology is still rapidly evolving and technical developments including scalability and parallelization have been making progress over the last years (Dawji et al., 2021), so that its broader application can be anticipated.

Finding circular elements in long read sequencing data can be considered as a pure assembly problem or as a special case of general structural variant calling from reads mapped to a reference genome, where (any combination of) duplications, (large) deletions, inversions or translocations are identified. This paper introduces a read mapping based approach to detect ecDNA in tumor cells. Technically, this is facilitated by enriching for circular DNA structures as linear DNA is digested during library preparation. Thus, the tasks can be defined as follows: 1) to identify reads that are part of circular DNA fragments and discriminate them from mapping errors and other ambiguous sequences; 2) to define fusions, breakpoints and boundaries in circular DNA by identification of split reads.

## 2 Methods

### 2.1 Sample Collection and Sequencing

#### 2.1.1 Cell Culture Conditions

Kelly cells (DSMZ Cat# ACC-355, RRID:CVCL_2092), which are derived from a human neuroblastoma that had high-level amplification of the MYCN oncogene, were obtained from the Leibniz Institute DSMZ (German Collection of Microorganisms and Cell Cultures, RRID:SCR_001711) and were cultivated in RPMI 1640 (Gibco, Paisley, United Kingdom) supplemented with 10% FBS, 1% Penicillin-Streptomycin (Gibco) and 2 mM l-glutamine (Gibco).

#### 2.1.2 Enrichment of Circular DNA

High molecular weight DNA was isolated from 1 × 10^6^ Kelly cells using the MagAttract HMW DNA Kit (QIAGEN, RRID:SCR_008539) according to the manufacturer’s protocol. DNA content was quantified using a NanoDrop Lite Spectrophotometer (Thermo Fisher Scientific, RRID:SCR_008452) and a Qubit 3.0 Fluorometer (Thermo Fisher Scientific). Exonuclease digestion of 5 µg high molecular DNA was performed with 20 units of the Plasmid-Safe ATP-Dependent DNase (Lucigen, Middleton, United States), 100 nmol of ATP (Lucigen), 10 µL of Plasmid-Safe 10x Reaction Buffer (Lucigen), and nuclease-free water in a total volume of 100 µL. The DNA was digested for 5 days at 37°C. Every 24 h additional 20 units of the Plasmid-Safe ATP-Dependent DNase, 100 nmol of ATP and 0.6 µL of Plasmid-Safe 10x Reaction Buffer were added to the reaction. After 5 days of digestion, the exonuclease was inactivated at 70°C for 30 min. The remaining circular DNA was amplified using the *φ*29 DNA polymerase supplied with the REPLI-g Mini Kit (Qiagen) according to manufacturer’s instructions.

For experiments involving recovery of circular plasmids, pDONR223_MYC_WT (RRID:Addgene82927) and ALK_pLenti (recombined plasmid of pLenti6.3/V5-DEST™, V53306, Thermo Fisher Scientific and pDONR223-ALK, RRID:Addgene23917) were mixed in the same ratio and amplified as described above.

Sample clean-up was performed using AMPure XP Beads (Beckman Coulter, RRID:SCR_008940) in a sample-beads ratio of 1:1.73. The beads were washed twice with 80% ethanol and DNA was eluted with 58 µL of distilled, nuclease-free water. A more detailed step-by-step protocol for circular DNA enrichment was published on the *Nature* Protocol Exchange server (Henssen et al., 2019). Before library preparation, the amplified samples were digested with T7 endonuclease I (New England Biolabs, RRID:SCR_013517) to remove branching. Digestion was performed for 15 min at 37°C using 15 units T7 endonuclease I, 3 µL NEBuffer 2, 1.5 µg amplified DNA, and nuclease-free water in a total volume of 30 µL. Longer fragments were selected using AMPure XP beads in a sample-beads ratio of 1:0.7. The beads were washed twice with 70% ethanol and DNA was eluted with 50 µL of nuclease-free water.

#### 2.1.3 Library Preparation and Sequencing

Samples enriched for ecDNAs were barcoded using EXP-NBD104 & SQK-LSK109 or SQK-RBK004 kits (Oxford Nanopore Technologies) according to the manufacturer’s protocol. Upon barcoding and ligation, samples were loaded onto a FLO-MIN106 (R9.4.1) flow cell and sequenced using a MinION (RRID:SCR_017985) (Oxford Nanopore Technologies, RRID:SCR_003756) for 48 h. After 24 h, the flow cell was flushed with the EXP-WSH003 wash kit and the multiplexed sample was reloaded onto the flow cell to increase the number of reads. An overview of the read length distributions for the samples are included in Supplementary Figures S3, S4.

#### 2.1.4 Validation of Predicted ecDNA in Kelly Cells

A predicted circle containing the MYCN gene was validated using PCR, gel electrophoresis and Sanger sequencing. Primers were designed using Primer Blast (Primer-BLAST, RRID:SCR_003095) and a 2 kb sequence upstream and downstream of the predicted breakpoint (5′-3′ FW: AGC​CAA​ACA​CAG​ACA​CAC​CA, RV: GGC​GGG​CCA​CTT​CAT​TAC​TT). DNA oligonucleotides were obtained from Integrated DNA Technologies (Coralville, United States). The PCR mix contained 10 µL MyTaq Polymerase (Meridian Bioscience, Memphis, United States), forward and reverse primer (20 µM) - each 1 μL, 3 µL nuclease-free water, and 5 µL of circular enriched DNA. PCR cycling conditions were adapted from the standard *MyTaq* protocol. Initial denaturation was performed for 5 min at 95°C followed by 35 cycles of denaturation (30 s at 95°C), annealing (30 s at primer specific annealing temperature), and extension (30 s at 72°C). Final extension was conducted for 10 min at 72°C. Gel electrophoresis was performed at 120 V for 45 min using an agarose gel (1%) supplemented with 1:10,000 GelRed (Biotium inc., RRID:SCR_013538). Afterwards, the amplicon was extracted from the gel with the Gel DNA Recovery Kit (Zymo Research, RRID:SCR_008968) and sent to Microsynth Seqlab GmbH (Göttingen, Germany) for Sanger sequencing using the forward primer.

### 2.2 *In Silico* Detection of Circular DNA Fragments

The overarching concept of our workflow was to first build a directed graph inferred from read depth information and split reads and then to identify plausible circular paths through its strongly connected components. Using samples that were sequenced as described in Section 2.1.1, read depth is expected to be almost zero at all loci that are not part of circular fragments, but strictly positive at loci that are part of circular fragments.

#### 2.2.1 Preprocessing

To construct the graph, information about read depth and split-reads is required. Therefore, reads are mapped using minimap2 (Minimap2, RRID:SCR_018550) (Li, 2018) and the human hg38 database as reference. Only for the recovery of plasmids as described below, the two plasmid sequences, pDONR223_MYC and pLenti_ALK were added as additional contigs. Read depth information is estimated from the mapping, similar to the method used by mosdepth (mosdepth, RRID:SCR_018929) (Pedersen and Quinlan, 2018) to solve the following task: For each mapped read, find its start and (calculated) end position in the reference, then increment a counter at its start position and decrement a counter at its end position. The cumulative sum of all reference position counters then gives an estimate of the read depth at each reference position. In order to allow mappers other than minimap2, split-reads are determined using standard SAM features only^1^, i.e. supplementary flag and SA tag.

#### 2.2.2 Building a Graph

We define a directed graph *G* = (*V*, *E*), where nodes *V* represent genomic loci and edges *E* ⊂ *V* × *V* between nodes carry additional information about the (mean) read depth and/or the number of split reads between them. A genomic position is in *V* if and only if there is a change in read depth at that locus, or if it is the start- or endpoint of a split read. An edge between two nodes exists if and only if the corresponding loci are neighbours in the contig or are connected by at least one split read. Hence, an edge may be of one of the following types:

• *coverage*: an edge between neighbouring nodes; the mean read depth in the genomic interval represented by the edge is at least θ∈N (default: *θ* = 1).
• *deletion*: an edge between neighbouring nodes; no reads cover the genomic interval represented by the edge.
• *split*: an edge between two nodes connected by at least one split-read.
• *coverage + split*: simultaneously *coverage* and *split* edge.

**FIGURE 1:**
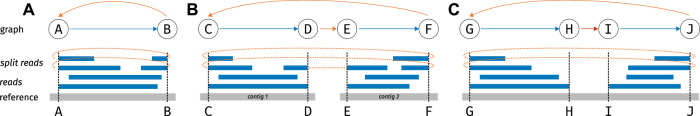
Example mappings and resulting graphs. The lower section of each panel A, B, C depicts the mapping of reads to a reference; the upper section show the graph. In the graph, nodes correspond to genomic loci (possible breakpoints), blue arrows correspond to *coverage* edges, red arrows to *deletion* edges and orange arrows to *split* edges **(A)** a simple circle on one contig **(B)** a circle formed from two parts on different contigs **(C)** a circle on one contig with a deletion in the middle (deletions are allowed because of library preparation).

Figure 1 gives examples of graphs constructed from a read mapping.

#### 2.2.3 Obtaining Plausible Paths

**FIGURE 2:**
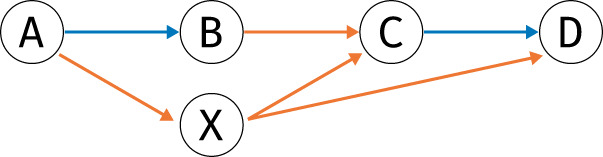
Example of a graph where pruning removes node X, since both incoming and outgoing edges are exclusively of type *split* (orange).

Once the graph has been built, the task is to find *plausible* circular paths: A path is deemed *plausible* if it alternates between *coverage* and either *split* or *deletion* edges. Every second edge has to be a coverage edge, because otherwise no genomic region on the reference would be covered. For example, looking at Figure 2, coverage edges *A* → *B* and *C* → *D* describe genomic ranges on the reference, while e.g. split edge *B* → *C* describes reads which *link* regions *AB* and *CD*, without actual coverage between loci *B* and *C*.

To find all plausible paths, we proceed as follows.

1. Prune nodes without incoming or outgoing coverage edges (such as node *X* in Figure 2). Because a plausible path alternates between coverage edges and other edges, any node that is not incident to a coverage edge cannot be part of a plausible path, and can hence be pruned from the graph.
2. Partition the graph into its strongly connected components (SCCs; in an SCC, each node can be reached from every other node). Circular paths are always part of a single SCC.
3. Enumerate each circular path in each SCC of the pruned graph.

The simplest plausible circular path consists of two nodes connected by *coverage + split* edges, defining a simple circle (or repeat) on the same contig (Figure 1A).

#### 2.2.4 Calling Events

For each plausible circular path, one candidate event is generated by translating *deletion* and *split* edges to a chain of breakends in VCF format^2^. Each of these candidate events is then called with varlociraptor (Köster et al., 2020) to obtain a posterior probability for each candidate to be truly present in the sample given the observed nanopore read data (using local re-alignments). With a set of circle calls *C* and a set of true circles *T* (i.e., the simulated circles), recall (which fraction of true circles was called?) and precision (which fraction of called circles are true circles?) are defined by

$$\text{recall}≔\frac{\left|C\cap T\right|}{\left|T\right|},\;\text{precision}≔\frac{\left|C\cap T\right|}{\left|C\right|}.$$

Our procedure makes use of varlociraptor’s ability to configure arbitrary statistical calling scenarios (beyond the initially published tumor/normal case in Köster et al. (2020)). The final annotated calls are output in tab-separated-value (TSV) format, and included into a final report for the user. The entire workflow is available at github.com/snakemake-workflows/cyrcular-calling. The easiest way to obtain and use it is via the snakemake workflow catalog: https://snakemake.github.io/snakemake-workflow-catalog?usage=snakemake-workflows/cyrcular-calling.

## 3 Results

### 3.1 Workflow for Detection of Circular DNA Fragments Using Nanopore Sequencing

**FIGURE 3:**
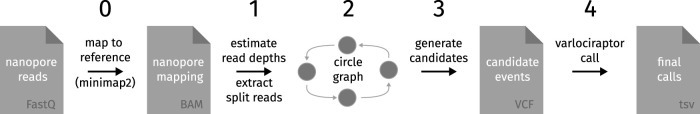
Steps for detection of circular DNA fragments **(0)** Mapping of nanopore reads to a reference genome using minimap2. **(1)** Estimation of read depth for each genomic locus and identification of split reads. **(2)** Building of a directed graph, where each node is a genomic locus and edges indicate split reads, covered segments or deletions. After inserting nodes and edges, the graph is pruned by removing nodes that only have split read edges. **(3)** For each strongly connected component, generate circular event candidates by enumerating *plausible* circular paths; a circular path is only plausible if every other edge is a coverage edge or if its length is two and it has two coverage and split read edges. **(4)** Call circular event candidates with varlociraptor. Calls from varlociraptor are then annotated with additional information and translated to tsv-format for the final report.

We implemented a Snakemake (Mölder et al., 2021) workflow for detecting circular DNA fragments from nanopore sequencing data using the protocol outlined in Section 2.1; see Figure 3. This workflow uses a set of nanopore reads in FastQ format to produce a table of highly likely circular events with relevant annotations, including probability of the event being present, plots of both the circular structure and detailed coverage of the event, genes covered by the event, and hyperlinks to primer-blast with the breakpoint sequence pre-filled to facilitate designing primers for wet-lab validation.

### 3.2 Evaluation of the Workflow Using Simulated ecDNA Reads

**FIGURE 4:**
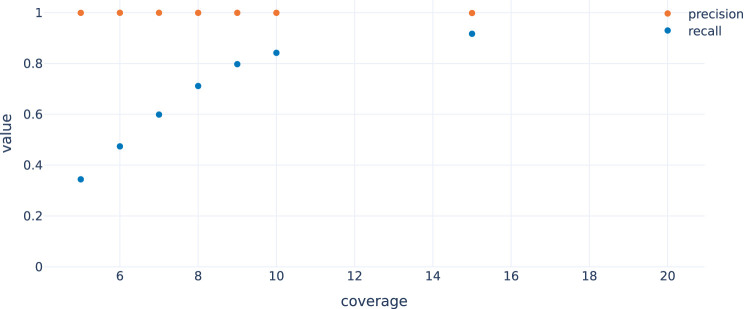
Recall and precision by coverage for data simulated as described in the main text. Recall improves with increasing coverage while precision is optimal independent of the coverage.

In order to evaluate the approach, we randomly selected 4995 genes (stratified by chromosome, maximum length = 1 Mbp, supplementary file simulation_regions.tsv) from which circular nanopore reads were simulated with nanosim (NanoSim, RRID:SCR_018243) (Yang et al., 2017) at a coverage of 25x. To evaluate performance also at lower coverages (5x–10x and 15x; see Figure 4), reads were subsampled from the 25x samples. To account for noise generated from non-circular DNA that remains after the preparation, we added simulated whole genome nanopore reads at 1x coverage. The simulated reads were then called using the workflow described in this paper. Figure 4 shows that recall increases with coverage as expected, since it gets more likely to observe reads spanning the fusion junction of a circle with increasing coverage (and noisy or erroneously or ambiguously mapping reads have less impact). Note that precision is always very close to 1, indicating that it is very unlikely to fabricate circles not in *T* with the chosen approach. This workflow is also suited to cope with short-read data, at least in principle. Using simulated Illumina data, we again find very high precision but a lower recall rate at comparable read depth (Supplementary Figure S5).

### 3.3 Model Validation to Recover Plasmids and to Detect the Mitochondrial Genome in Tumor DNA

**FIGURE 5:**
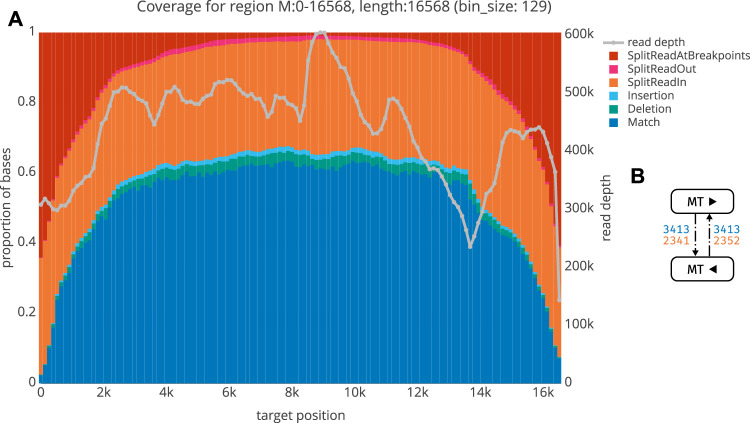
A QC plot for the mitochondrion from Kelly sample. For each locus (bin), we obtain a summary of the number of matches (blue), insertions (cyan) and deletions (green) as well as the number of bases belonging to a split read either within the region (orange), spanning over both breakpoints (red), or linking to some other region (magenta). On the second *y*-axis, read depth is shown (grey) **(B)** Graph structure for the Kelly mitochondrion, with the same keys as in Figure 6.

**FIGURE 6:**
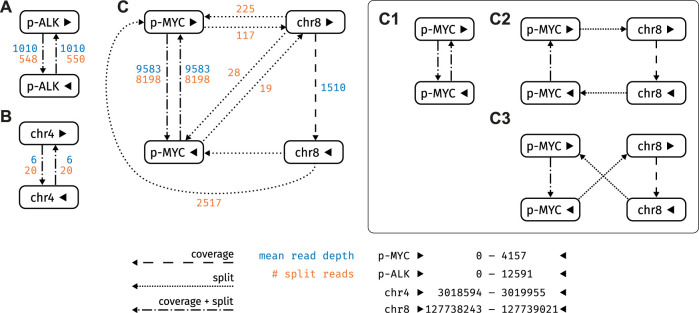
Graph structure of the strongly connected components (SCC) of the plasmid sample. Nodes refer to reference loci, edges as described in Section 2.2.2 **(A)** SCC containing ALK_pLenti (posterior probability 1.0) **(B)** SCC with an off-target hit on chromosome 4 (posterior probability 0.0) **(C)** SCC containing pDONR223_MYC_WT: This SCC encompasses three plausible circular paths **(C1) (C2)** and **(C3)**, all of which are equally likely (posterior probability 1.0).

We next aimed to validate our workflow using plasmid controls and human tumor cell DNA. The mitochondrial genome was used as an internal positive control, since it is circular and is hence selected for during ecDNA library preparation. Moreover, it does not share extensive homology with human chromosomal DNA. Using ecDNA isolated from human neuroblastoma cell lines (Kelly cells; Section 2.1.1), we indeed find that the mitochondrial genome is recovered as circular DNA. Figure 5 shows an exemplary quality control plot of the mitochondrial genome in these cells. Basically, all circular segments are characterized by a high proportion of split reads at breakpoints and the proportion of split reads gradually decreases towards the middle. As not all split reads start or end exactly at breakpoints (due to noisiness, mapping issues etc), we termed those split reads that start or end up to 3bp up- and/or downstream of the breakpoint as *SplitReadsAtBreakpoints* (Figure 5). Modifying this value will shift a portion of the split reads from *SplitReadIn* to the *SplitReadsAtBreakpoints* category. The number of inner-split-reads in the different categories can be modulated via the threshold for mapping quality (MAPQ, exemplarily shown for MAPQ ≥0 and MAPQ ≥60 in Supplementary Figures S6, S7, respectively). Notably, these settings only affect the visualization, while the statistical assessment of the candidate circles in Varlociraptor works via pair-HMM based realignment of all reads that overlap the breakpoints. Next, we devised an experiment in which two plasmids, pDONR223_MYC_WT and ALK_pLenti, containing cDNA coding for the MYC and the ALK gene, respectively, were sequenced. As the plasmid sequences do not occur in the reference genome, we added them as separate contigs to the hg38 reference for mapping purposes. Nanopore sequencing of this sample with subsequent application of our workflow allowed for recovery of both plasmids, however, additional putative circles were detected (Figure 6). Here, statistical calling with Varlociraptor correctly discarded an off-target hit on chromosome 4 (Figure 6B), while homology of the MYC plasmid to the chromosomal region in the vicinity of the MYC gene gave rise to three plausible circular paths, one of which describes only the plasmid, while the other two contain a stretch on chromosome eight encompassing the MYC locus. Due to extensive homologies, the alternative hits for the MYC plasmid were considered equally likely (Figure 6C).

### 3.4 Defining the Boundaries of Circular DNA Encompassing the MYCN Oncogene in Human Neuroblastoma Cells

**FIGURE 7:**
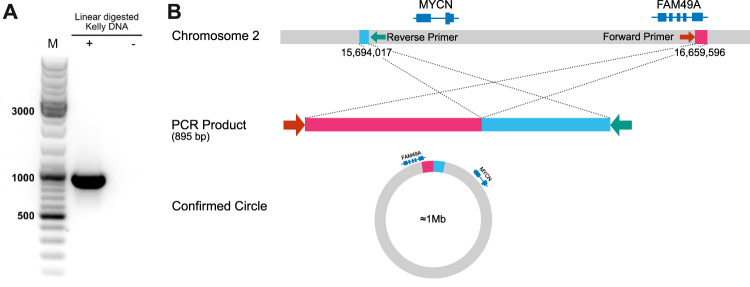
Detection of an extracellular amplicon encompassing the MYCN oncogene in the cell line Kelly **(A)** Gel electrophoresis of ecDNA fragment, which was amplified by primers flanking the breakpoint **(B)** Schematic representation and interpretation of Sanger sequencing results.

**FIGURE 8:**
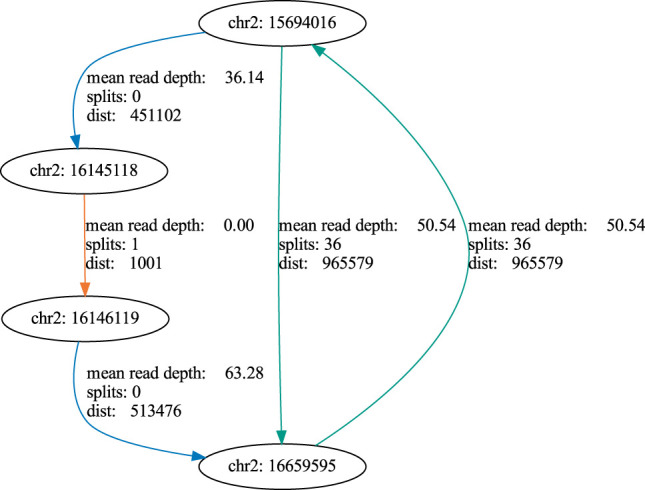
Graph of an extracellular amplicon encompassing the MYCN oncogene in the cell line Kelly. blue: coverage edge, orange: split edge, green: coverage + split edge.

In order to address our goal of identifying tumor cell-specific ecDNA by Nanopore sequencing, we made use of the human neuroblastoma cell line, Kelly. A subset of neuroblastoma is characterised by genomic amplification of the MYCN oncogene. These DNA fragments are designated as double minutes (DMs) when occurring extrachromosomally. Kelly cells are known to carry extra copies of the MYCN-coding gene on these DMs (Helmsauer et al., 2020). We thus set out to validate our workflow by recovery of MYCN-containing DNA circles in Kelly cells and to define their boundaries. Indeed, nanopore sequencing of ecDNA from Kelly cells revealed a 964,578 bp circular element spanning the region 15 694 017–16 659 596 of chromosome 2 (Figure 8, Supplementary Figure S1), which encompasses the genomic locus of the MYCN gene as well as the FAM49A locus. To validate this finding by an orthogonal method, we again used ecDNA isolated from Kelly cells in a PCR reaction together with a forward primer binding to 16 659 067–16 659 086 and a reverse primer binding to 15 694 363–15 694 382 on chromosome 2. We successfully obtained a PCR product of the predicted length at the circle junction (Figure 7A). Sanger sequencing of the amplified DNA fragment verified that this PCR product in fact perfectly aligned with the MYCN gene and confirmed the localization of the breakpoint (Figure 7B, Supplementary Figure S2). See supplementary data file example_report.zip for the report generated by the workflow.

## 4 Discussion

Extrachromosomal circularization of DNA is a common event in cancer cells and is associated with poor patient outcome. Thus, ecDNAs could serve as potential biomarkers to monitor disease progression. Here, we describe a computational ecDNA detection workflow which uses nanopore sequencing reads derived from DNA samples enriched for circular DNA. Our workflow enables the detection of circular DNA with arbitrary fusions, even if they originate from different chromosomes. While we designed our approach with nanopore sequencing data (and long reads in general) in mind, it can be applied to short read data as well. Here, similar to nanopore data, the precision remains constantly high regardless of the coverage. By contrast, the achieved recall is generally lower when using Illumina data only (see Subsection 3.2, Supplementary Figure S5). This might improve by parameter tuning and including read pair evidence in the candidate circle detection in future work. These improvements, however, still had to cope with the intrinsic limitations of short-read sequencing, e.g. in highly repetitive regions.

In general, there are two obvious options for detection of circular DNA in long read sequencing data: read mapping or *de novo* assembly. Here, we have chosen a read-based mapping approach. This enables us to use the variant calling model of Varlociraptor to assess the read support for each candidate circle statistically, under consideration of all involved uncertainties about mapping location and alignment ambiguities. Hence, we can obtain a posterior probability for the existence of each candidate circle and control the local false discovery rate. Moreover, it in theory allows to correlate results from nanopore sequencing and short read Illumina data for improved statistical power and robustness, as it has been shown by previous publications (Kim et al., 2020; Koche et al., 2020). In our case, it would be possible to use Varlociraptor’s variant calling grammar^3^ to calculate the posterior probability that a circle is present in both the Illumina and the Nanopore sample. The downsides of the mapping approach are that it is susceptible to mapping artifacts not captured by mapping quality (MAPQ) or mapping ambiguities. Moreover, it is cumbersome to integrate unknown sequences not present in the reference. On the other hand, assembly based approaches suffer from the difficulty to handle the repetitive nature of circles (i.e. assembling too many runs through the circle), and there is currently no statistical approach for quantifying the uncertainty of results obtained by this method.

Using this workflow we first demonstrated seamless recovery of the mitochondrial (mt) genome, which is a ubiquitous source of circular DNA in human cells. The mt genome can thus serve as a positive control when analyzing ecDNA against the background of the entire humane genome. We also showed that our workflow is also capable of recovering circular plasmid DNA. Thus, combining the computational workflow and the experimental setup equipped us with a powerful tool to detect ecDNA by nanopore sequencing data in human tumor cells. We next went on to apply our workflow to a more clinically relevant problem, which is detection of ecDNA from tumor cells. For this purpose, we used ecDNA from human neuroblastoma cells, Kelly, to map and recover the entire 966 kb extrachromosomal MYCN oncogene amplicon (Figure 8). Additionally, we were able to map the chromosomal localization of the breakpoint within this amplicon. Again, this validates that our workflow robustly works over a wide range of DNA fragment sizes to detect ecDNAs by nanopore sequencing. Even more important, this notion points to future applications of our workflow: As ecDNA is specific to tumor cells and is associated with aggressive disease courses, ecDNA detection could be used as a patient-specific fingerprint for early detection of solid tumors that have a high risk of recurrence. For this purpose, ecDNA obtained from a tumor sample at diagnosis could be used to define a DNA fragment that can be detected by PCR, which is sensitive enough to identify the presence of a few tumor cells harboring the ecDNA against the background of the normal DNA of the patient. Detecting tumor-specific DNA in blood is already common clinical practice in cancer patients with known mutational profiles (e.g. EGFR-mutant lung cancer) and therapy will be adapted according to presence or absence of certain tumor-specific mutations. By contrast, detection of ecDNA had the advantage of being agnostic to mutational profiles that occur only in a fraction of tumor patients. Thus, ecDNA detection could be individually adapted for those tumor types, in which ecDNA are most common such as brain tumors, lung tumors and others (Kim et al., 2020). Tracking ecDNAs might be highly beneficial as an early-warning system informing on failure of therapies and thus help to optimize sequential therapies of patients with solid tumors.

## Data Availability

The datasets presented in this study can be found in online repositories. The names of the repository/repositories and accession number(s) can be found below: https://www.ebi.ac.uk/ena, PRJEB50518.

## Footnotes

1 see https://samtools.github.io/hts-specs/SAMv1.pdf and https://samtools.github.io/hts-specs/SAMtags.pdf

2
https://samtools.github.io/hts-specs/VCFv4.2.pdf

3
https://varlociraptor.github.io/docs/calling#generic-variant-calling
