# Supplementary material for: Detection and Validation of Circular DNA Fragments Using Nanopore Sequencing
Source: Front Genet. 2022 May 30;13:867018. doi: 10.3389/fgene.2022.867018 (PMC9195511; doi:10.3389/fgene.2022.867018)
Supplement: Supplementary file 1 [file Presentation1.pdf]

Supplementary Material

1 SUPPLEMENTARY DATA

The supplementary file `example_report.zip` contains an exemplary interactive report as generated by the cyrcular workflow. The initial view displays the workflow's rules and their dependencies on each other. The "Statistics" view gives information about the run time of every single rule application. For example, the circle calling step (`calling_circle_bnds`) takes 76s for the kelly sample and 345s for the pLenti sample. The workflow was executed on a machine with an AMD Opteron™ Processor 6176 (48 cores, 500GB RAM). The "Circle calls" view lists one interactive table for each sample processed with the workflow; the table column `EVENT` contains links to QC plots of the respective events.[1]

2 SUPPLEMENTARY TABLES AND FIGURES

2.1 Figures

[1] Note that each event requires multiple rows, at least two for each breakpoint. For example, the Mitochondrion of the Kelly sample is a simple circle with one breakpoint, which is encoded as two rows.

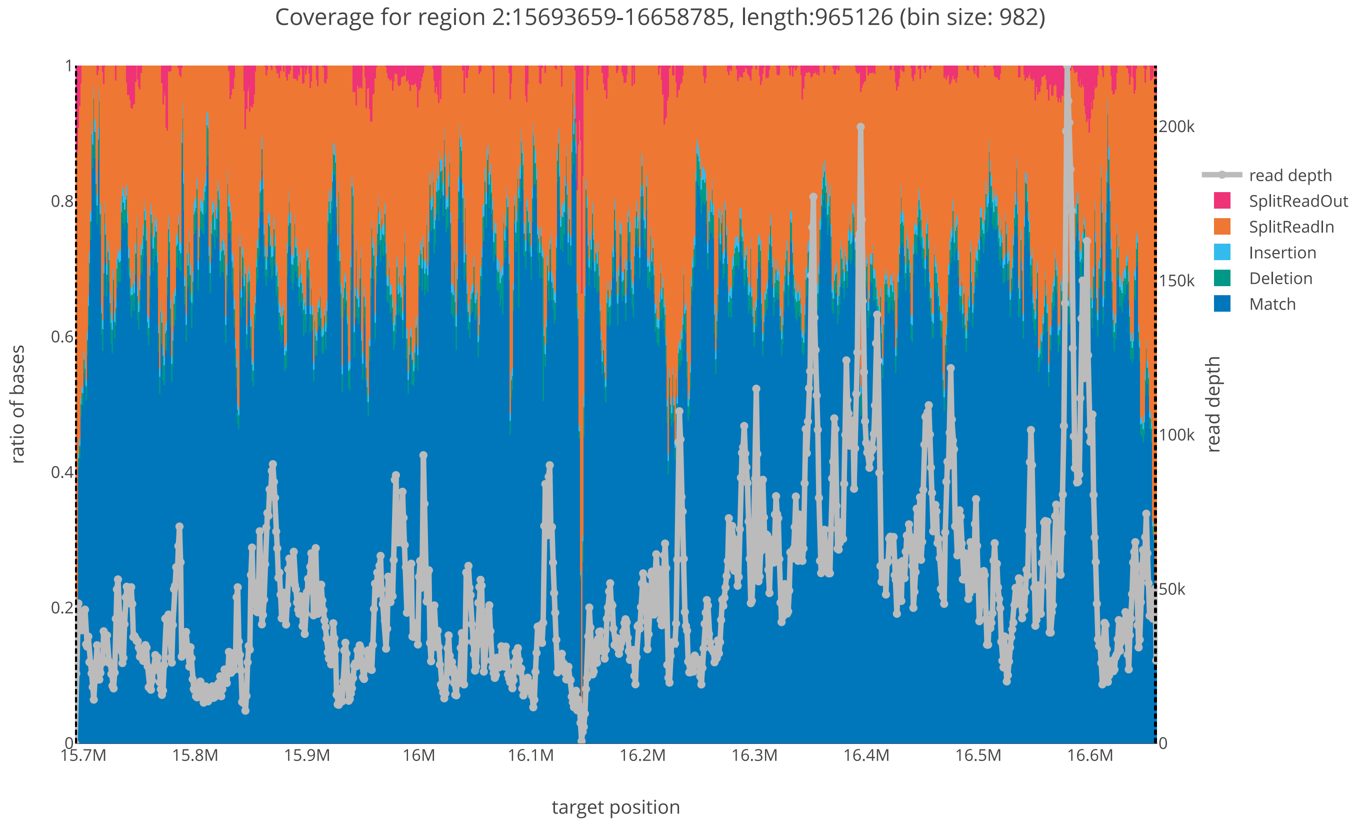

Figure S1. QC plot for the MYCN circle of the kelly sample. For each locus (bin), we obtain a summary of the number of matches (blue), insertions (cyan) and deletions (teal) as well as the number of bases belonging to a split read either within the region (orange) or linking to some other region (magenta). On the second y-axis, read depth is shown (grey).

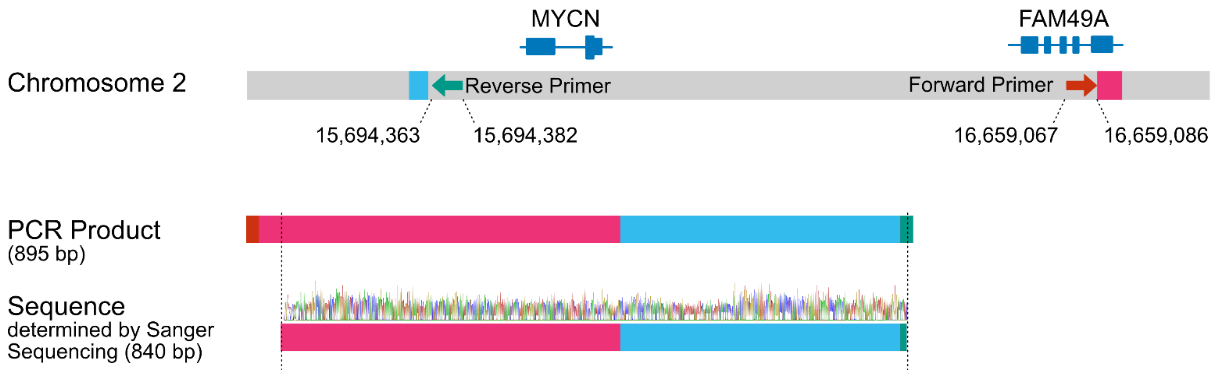

NCBI Blast results of sequenced PCR product (Aligned to *Homo sapiens chromosome 2, GRCh38.p13 Primary Assembly*)

Query 1<br>Sbjct 16,659,110	CAAGCTATATTTCATTCAGTTCTAAGGTATGGAGGCTCCTCTGGCTAGCAGAAACTCACC
Query 61<br>Sbjct 16,659,170	AGCTCTTTGAGATGAGTGTTCTACCCAAGACACATGCTAATTAACTCCATGCTCCTCACA
Query 121<br>Sbjct 16,659,230	TATGCACAGCTCTTGTTTATAGTGTGGGTGATACAGAGAGATCAGGCTTTGAAGGCACAC
Query 181<br>Sbjct 16,659,290	AGATGTGGTTTCAGATACCAGCTATGCCACTTTTCTAATGAGGTGACATTCTGCAAATTC
Query 241<br>Sbjct 16,659,350	CTTAACACCTCTGGACTTCCATTCGCCCATCCATAAAATGGGGATAATATCTGTTTCAAA
Query 301<br>Sbjct 16,659,410	CGTTCTTCAGAAGATTGCTAAAAGTGGGTACAAAATGTTTATCACTGTCCTGCTGACAAT
Query 361<br>Sbjct 16,659,470	ACCCTACATGGCTCAGTAGGAACACAAGACTCAGGAGATCGACAGCTTGTCAAAGCCCAG
Query 421<br>Sbjct 16,659,530	GCTGCTTGTAAGTGGGCAGCGTGACTACAAGACCCCGGGTCTTCTTGGACTCTCCACTTG
Query 481<br>Sbjct 16,659,590	CTCTTGC
Query 487<br>Sbjct 15,694,017	TCTTTACTTTCTGCTCAGTTTTGCTGTGAACCTAAAACTACTGCAAAAAAATAAAAAATA
Query 547<br>Sbjct 15,694,077	GAAAAGACTCAGACTTTAATTACAGAAGAGAAGCTTTCCCTTACCCCAGCTTGGCAGATT
Query 607<br>Sbjct 15,694,137	CTGCTGAGGGGCCGGGGTCTCCTTGGAGAGGCAGCCTTGGACAGCTGGGGGTCCCCGCTC
Query 667<br>Sbjct 15,694,197	CCCACCCCAGTCTCAGGCCGCGCCCGGGCTGGCTGTGTGGAGAGACGTCTGTTGTGGATC
Query 727<br>Sbjct 15,694,257	CCCCACACTACTTTCGCTTGCTATAAAGCAACACTGGGAGTGAATTTAGTGGTTGTGAAA
Query 787<br>Sbjct 15,694,317	TTTAAAAAGGCAAGTGCAGAGCTGGGAGGCATTTCCTTAAGCACAAAGTAATG

Figure S2. Sanger sequencing results and Blast output for sequenced PCR product.

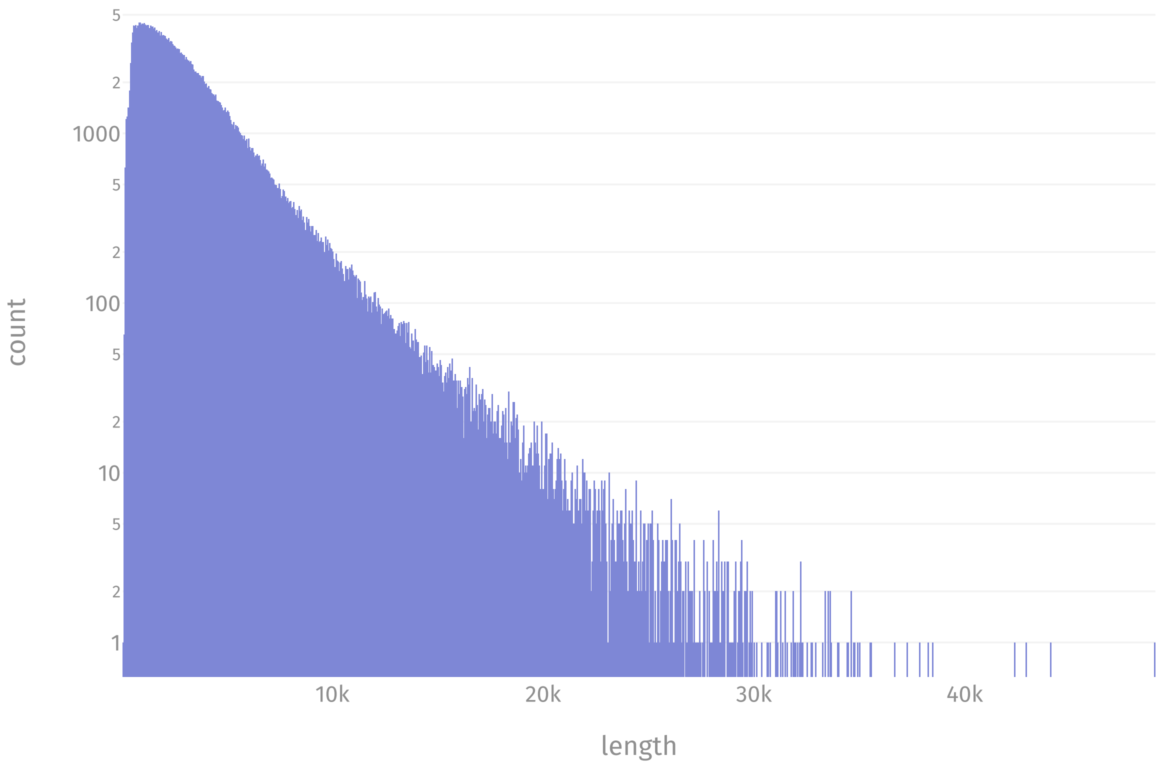

Figure S3. Read length histogram for the Kelly sample. The median (read length) is 2551, mean is ∼3374, standard deviation is ∼2984.

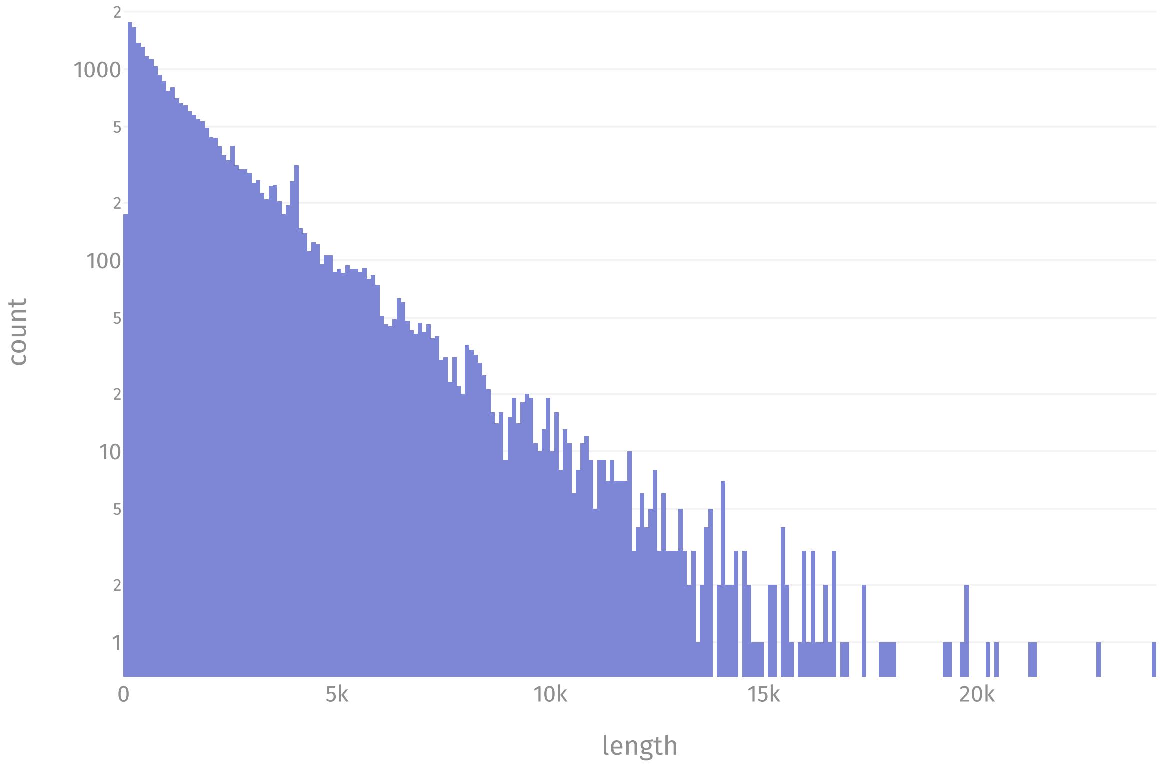

Figure S4. Read length histogram for the (downsampled) pLenti sample. The median (read length) is 1294, mean is ∼2018, standard deviation is ∼2174.

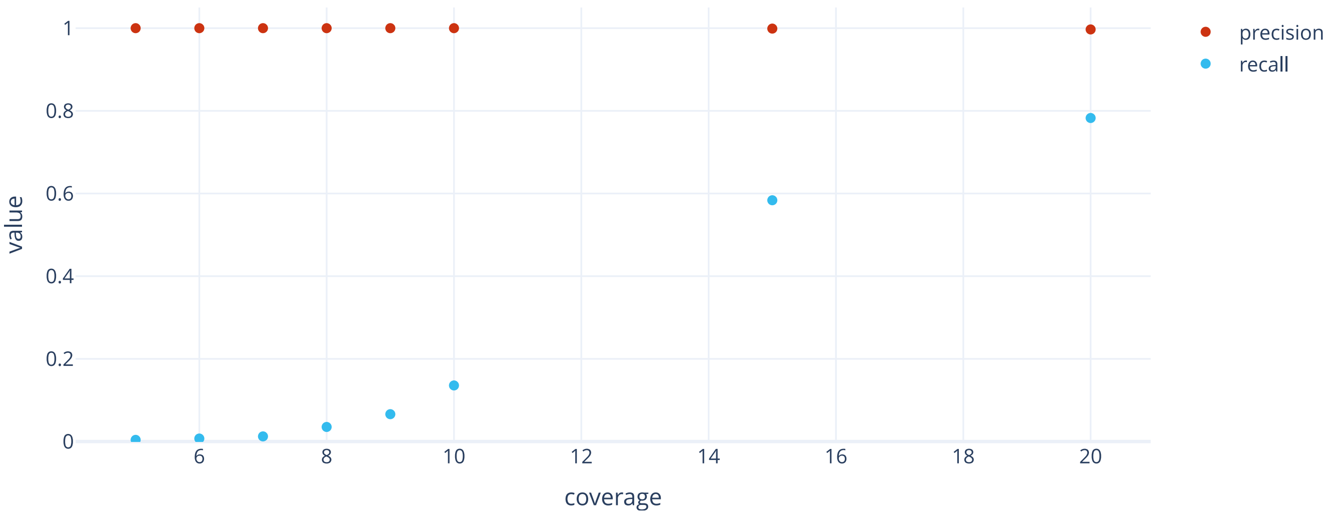

Figure S5. Precision and recall obtained by running the validation workflow on an experiment where Illumina 100bp (HiSeq 2000) circular reads and non-circular WGS reads were simulated (same as for the nanopore simulation, but with `readSimulator.py` instead of `nanoSim`). This performs significantly and consistently worse than for nanopore data on all coverages tested.

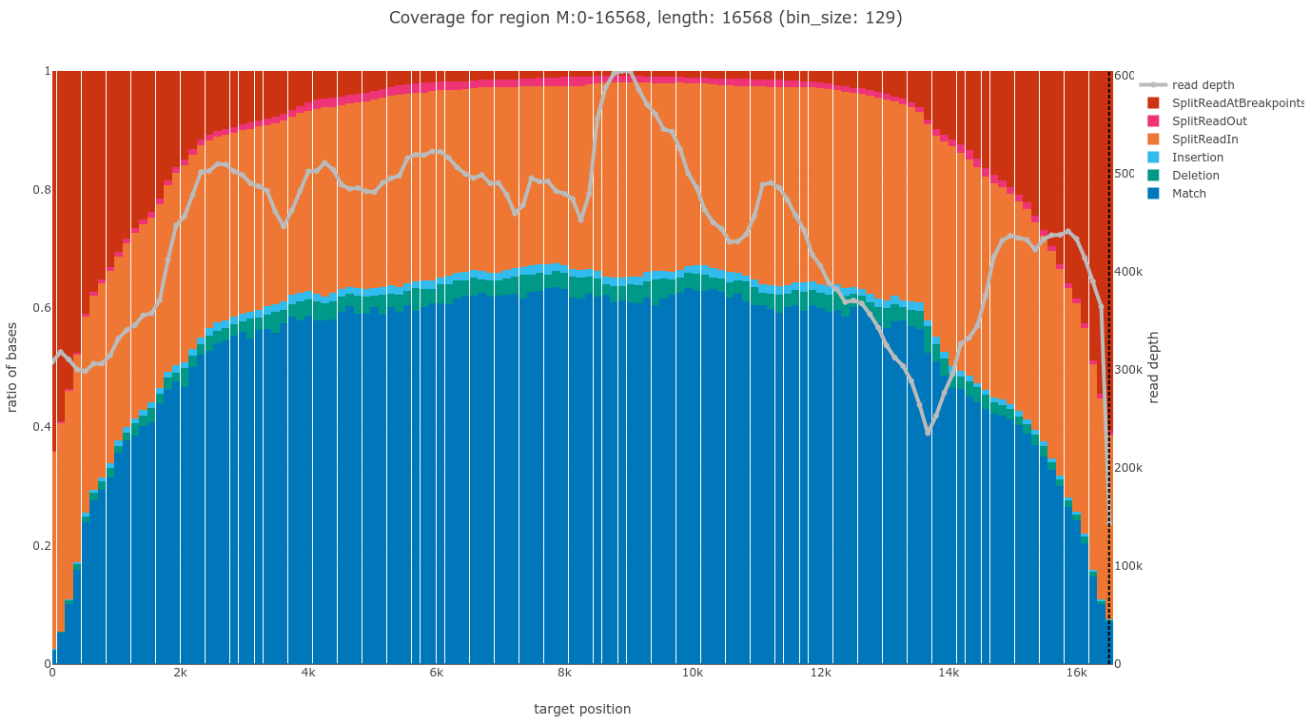

Figure S6. QC plot for the Kelly sample (see fig. 5A in main manuscript) for reads with mapping quality ≥ 0

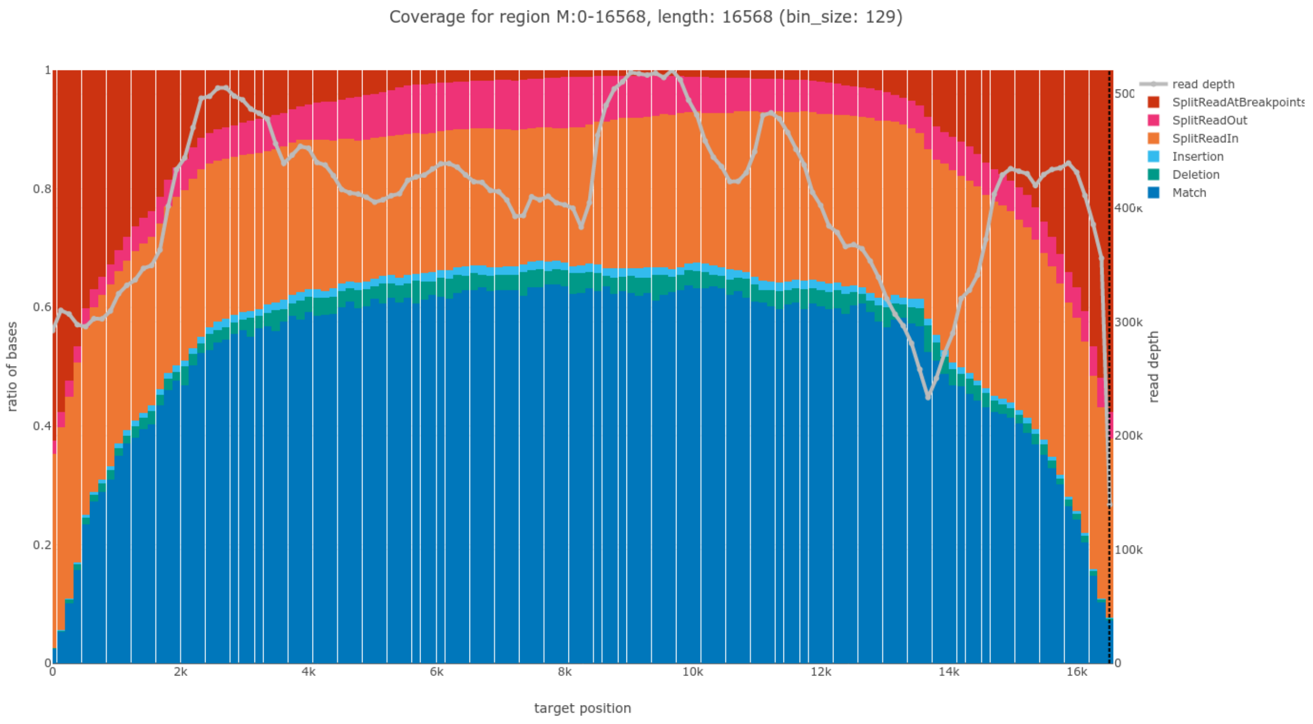

Figure S7. QC plot for the Kelly sample (see fig. 5A in main manuscript) for reads with mapping quality ≥ 60
