# Supplementary material for: Detection and Validation of Circular DNA Fragments Using Nanopore Sequencing
Source: Front Genet. 2022 May 30;13:867018. doi: 10.3389/fgene.2022.867018 (PMC9195511; doi:10.3389/fgene.2022.867018)
Supplement: Supplementary file 2 [file DataSheet1.ZIP › example_report/report.html]

Snakemake Report


Loading Snakemake Report...

Please enable Javascript in your browser to see this report.

Snakemake Report

- Fri Jan 28 10:40:03 2022 UTC
- Snakemake 6.12.3

- Workflow (current)
- Statistics
- Configuration

##### Results

- Circle calls


## Workflow

Click the nodes to obtain details about each step.

## Circle calls

## Statistics

If the workflow has been executed in cluster/cloud, runtimes include the waiting time in the queue.

## Configuration

Configuration files

| File | Code |
| --- | --- |
|  | |  |  | | --- | --- | | ```  1  2  3  4  5  6  7  8  9 10 11 12 13 14 15 16 17 18 19 20 21 22 23 24 ``` | ``` calling:   samples: config/samples.tsv   units: config/units.tsv    reference: #     name: "grch38_with_plasmid_replacements" #     path: "resources/grch38_with_plasmid_replacements.fasta"     name: "grch38_with_alk_myc_plasmids"     path: "resources/grch38_with_alk_myc_plasmids.fasta"    min_read_depth: 2   min_split_reads: 5   max_paths_per_component: 15   max_deletion_length: 10000    # TODO: actually use these settings   filter:     fdr-control:       threshold: 0.05       local: true       events:         circular:           varlociraptor:             - present ``` | |

Loading...

##### 

×

Download original

##### Rule calling\_annotate\_breakpoint\_sequence

×

Rule properties

|  |  |
| --- | --- |
| Jobs | 2 |
| Input files | |
| - results/calling/tables/{group}.sorted.csv - resources/grch38\_with\_alk\_myc\_plasmids.fasta | |
| Output files | |
| - results/calling/tables/{group}.sorted.annotated.csv | |
| Conda software stack | |
| - pandas >=1.3.4 - pandarallel >=1.5.4 - dinopy >=2.2.0 | |

##### Rule calling\_sort\_and\_tidy\_tsv

×

Rule properties

|  |  |
| --- | --- |
| Jobs | 2 |
| Input files | |
| - results/calling/tables/{group}.tsv | |
| Output files | |
| - results/calling/tables/{group}.sorted.csv | |
| Conda software stack | |
| - pandas >=1.3.4 - pandarallel >=1.5.4 - dinopy >=2.2.0 | |

##### Rule calling\_vembrane\_table

×

Rule properties

|  |  |
| --- | --- |
| Jobs | 2 |
| Input files | |
| - results/calling/calls/filtered/{group}.bcf | |
| Output files | |
| - results/calling/tables/{group}.tsv | |
| Conda software stack | |
| - vembrane =0.7 - bcftools =1.12 | |
| Code | |
| |  |  | | --- | --- | | ``` 1 ``` | ``` vembrane table {params.extra} '{params.expression}' {input.vcf} > {output.tsv} ``` | | |

##### Rule calling\_filter\_calls

×

Rule properties

|  |  |
| --- | --- |
| Jobs | 2 |
| Input files | |
| - results/calling/calls/annotated/{group}.bcf | |
| Output files | |
| - results/calling/calls/filtered/{group}.bcf - results/calling/calls/filtered/{group}.at\_least\_one\_exon.bcf | |
| Conda software stack | |
| - vembrane =0.7 - bcftools =1.12 | |
| Code | |
| |  |  | | --- | --- | | ``` 1 2 3 ``` | ```         vembrane filter '{params.filter_expression}' {input} -O bcf > {output.by_exons} 2> {log}         varlociraptor filter-calls control-fdr {params.mode} --events PRESENT --var BND --fdr {params.fdr} {output.by_exons} | varlociraptor decode-phred | bcftools sort -m 4G -Ob > {output.calls} 2>> {log} ``` | | |

##### Rule calling\_copy\_annotation\_from\_cyrcular

×

Rule properties

|  |  |
| --- | --- |
| Jobs | 2 |
| Input files | |
| - results/calling/calls/pre\_annotated/{sample}.bcf - results/calling/calls/pre\_annotated/{sample}.bcf.csi - results/calling/candidates/{sample}.sorted.bcf - results/calling/candidates/{sample}.sorted.bcf.csi | |
| Output files | |
| - results/calling/calls/annotated/{sample,[a-zA-Z\_0-9-]+}.bcf | |
| Conda software stack | |
| - bcftools =1.12 | |
| Code | |
| |  |  | | --- | --- | | ``` 1 2 ``` | ```         bcftools annotate --annotations {input.candidates_with_annotation} --columns {params.columns} --output-type b --output {output.variants} {input.variants} ``` | | |

##### Rule calling\_annotate\_genes

×

Rule properties

|  |  |
| --- | --- |
| Jobs | 2 |
| Input files | |
| - resources/gencode.v38.annotation.sorted.gff3.gz - results/calling/calls/merged/{sample}.bcf | |
| Output files | |
| - results/calling/calls/pre\_annotated/{sample,[a-zA-Z\_0-9-]+}.bcf | |
| Conda software stack | |
| - bcftools =1.13 - pysam - gff3sort | |

##### Rule sort\_annotation

×

Rule properties

|  |  |
| --- | --- |
| Jobs | 1 |
| Input files | |
| - resources/gencode.v38.annotation.gff3.gz | |
| Output files | |
| - resources/gencode.v38.annotation.sorted.gff3.gz - resources/gencode.v38.annotation.sorted.gff3.gz.tbi - resources/gencode.v38.annotation.sorted.gff3.gz.csi | |
| Conda software stack | |
| - bcftools =1.13 - pysam - gff3sort | |
| Code | |
| |  |  | | --- | --- | | ``` 1 2 3 4 ``` | ```         gff3sort.pl <(pigz -dc {input}) | bgzip -@ {threads} -c > {output.gff} 2> {log}         tabix {output.gff} 2>> {log}         tabix --csi {output.gff} 2>> {log} ``` | | |

##### Rule calling\_bcftools\_concat

×

Rule properties

|  |  |
| --- | --- |
| Jobs | 2 |
| Input files | |
| - results/calling/calls/initial\_sorted/{group}.1-of-12.bcf - results/calling/calls/initial\_sorted/{group}.2-of-12.bcf - results/calling/calls/initial\_sorted/{group}.3-of-12.bcf - results/calling/calls/initial\_sorted/{group}.4-of-12.bcf - results/calling/calls/initial\_sorted/{group}.5-of-12.bcf - results/calling/calls/initial\_sorted/{group}.6-of-12.bcf - results/calling/calls/initial\_sorted/{group}.7-of-12.bcf - results/calling/calls/initial\_sorted/{group}.8-of-12.bcf - results/calling/calls/initial\_sorted/{group}.9-of-12.bcf - results/calling/calls/initial\_sorted/{group}.10-of-12.bcf - results/calling/calls/initial\_sorted/{group}.11-of-12.bcf - results/calling/calls/initial\_sorted/{group}.12-of-12.bcf - results/calling/calls/initial\_sorted/{group}.1-of-12.bcf.csi - results/calling/calls/initial\_sorted/{group}.2-of-12.bcf.csi - results/calling/calls/initial\_sorted/{group}.3-of-12.bcf.csi - results/calling/calls/initial\_sorted/{group}.4-of-12.bcf.csi - results/calling/calls/initial\_sorted/{group}.5-of-12.bcf.csi - results/calling/calls/initial\_sorted/{group}.6-of-12.bcf.csi - results/calling/calls/initial\_sorted/{group}.7-of-12.bcf.csi - results/calling/calls/initial\_sorted/{group}.8-of-12.bcf.csi - results/calling/calls/initial\_sorted/{group}.9-of-12.bcf.csi - results/calling/calls/initial\_sorted/{group}.10-of-12.bcf.csi - results/calling/calls/initial\_sorted/{group}.11-of-12.bcf.csi - results/calling/calls/initial\_sorted/{group}.12-of-12.bcf.csi | |
| Output files | |
| - results/calling/calls/merged/{group}.bcf | |
| Conda software stack | |
| - bcftools =1.12 - snakemake-wrapper-utils ==0.2.0 | |

##### Rule calling\_bcftools\_sort

×

Rule properties

|  |  |
| --- | --- |
| Jobs | 24 |
| Input files | |
| - results/calling/calls/initial/{group}.{scatteritem}.bcf | |
| Output files | |
| - results/calling/calls/initial\_sorted/{group}.{scatteritem,\d+-of-\d+}.bcf | |
| Conda software stack | |
| - bcftools ==1.11 | |

##### Rule calling\_varlociraptor\_call

×

Rule properties

|  |  |
| --- | --- |
| Jobs | 24 |
| Input files | |
|  | |
| Output files | |
| - results/calling/calls/initial/{group}.{scatteritem,\d+-of-\d+}.bcf | |
| Code | |
| |  |  | | --- | --- | | ``` 1 ``` | ``` varlociraptor call variants generic --obs {params.obs} --scenario {input.scenario} > {output} 2> {log} ``` | | |

##### Rule calling\_varlociraptor\_preprocess

×

Rule properties

|  |  |
| --- | --- |
| Jobs | 24 |
| Input files | |
| - resources/grch38\_with\_alk\_myc\_plasmids.fasta - resources/grch38\_with\_alk\_myc\_plasmids.fasta.fai - results/calling/mapping/{sample}.bam - results/calling/mapping/{sample}.bam.bai | |
| Output files | |
| - results/calling/calls/observations/{sample,[a-zA-Z\_0-9-]+}.{scatteritem,\d+-of-\d+}.bcf | |
| Code | |
| |  |  | | --- | --- | | ``` 1 ``` | ``` varlociraptor preprocess variants {input.ref} --candidates {input.candidates} --model {params.model} --max-depth 200 --bam {input.bam} --output {output} 2> {log} ``` | | |

##### Rule calling\_scatter\_candidates

×

Rule properties

|  |  |
| --- | --- |
| Jobs | 2 |
| Input files | |
| - results/calling/candidates/{sample}.sorted.bcf | |
| Output files | |
| - results/calling/candidate-calls/{sample,[a-zA-Z\_0-9-]+}.1-of-12.bcf - results/calling/candidate-calls/{sample,[a-zA-Z\_0-9-]+}.2-of-12.bcf - results/calling/candidate-calls/{sample,[a-zA-Z\_0-9-]+}.3-of-12.bcf - results/calling/candidate-calls/{sample,[a-zA-Z\_0-9-]+}.4-of-12.bcf - results/calling/candidate-calls/{sample,[a-zA-Z\_0-9-]+}.5-of-12.bcf - results/calling/candidate-calls/{sample,[a-zA-Z\_0-9-]+}.6-of-12.bcf - results/calling/candidate-calls/{sample,[a-zA-Z\_0-9-]+}.7-of-12.bcf - results/calling/candidate-calls/{sample,[a-zA-Z\_0-9-]+}.8-of-12.bcf - results/calling/candidate-calls/{sample,[a-zA-Z\_0-9-]+}.9-of-12.bcf - results/calling/candidate-calls/{sample,[a-zA-Z\_0-9-]+}.10-of-12.bcf - results/calling/candidate-calls/{sample,[a-zA-Z\_0-9-]+}.11-of-12.bcf - results/calling/candidate-calls/{sample,[a-zA-Z\_0-9-]+}.12-of-12.bcf | |
| Code | |
| |  |  | | --- | --- | | ``` 1 ``` | ``` rbt vcf-split {input} {output} ``` | | |

##### Rule calling\_sort\_bnd\_bcfs

×

Rule properties

|  |  |
| --- | --- |
| Jobs | 2 |
| Input files | |
| - results/calling/candidates/{sample}.bcf | |
| Output files | |
| - results/calling/candidates/{sample,[a-zA-Z\_0-9-]+}.sorted.bcf | |
| Conda software stack | |
| - bcftools ==1.11 | |

##### Rule calling\_circle\_bnds

×

Rule properties

|  |  |
| --- | --- |
| Jobs | 2 |
| Input files | |
| - results/calling/mapping/{sample}.bam - results/calling/mapping/{sample}.bam.bai - resources/grch38\_with\_alk\_myc\_plasmids.fasta | |
| Output files | |
| - results/calling/candidates/{sample,[a-zA-Z\_0-9-]+}.bcf - results/calling/graphs/{sample,[a-zA-Z\_0-9-]+}.graph - results/calling/graphs/{sample,[a-zA-Z\_0-9-]+} | |
| Code | |
| |  |  | | --- | --- | | ``` 1 ``` | ``` cyrcular        graph        breakends        {input.bam}        --reference {input.ref}        --min-read-depth {params.min_read_depth}        --min-split-reads {params.min_split_reads}        --max-paths-per-component {params.max_paths_per_component}        --max-deletion-length {params.max_deletion_length}        -t 8        --output {output.bnds}        --graph {output.graph}        --dot {output.dot}        2> {log} ``` | | |

##### Rule calling\_minimap2\_bam

×

Rule properties

|  |  |
| --- | --- |
| Jobs | 2 |
| Input files | |
| - results/calling/index/grch38\_with\_alk\_myc\_plasmids.mmi | |
| Output files | |
| - results/calling/mapping/{sample,[a-zA-Z\_0-9-]+}.bam | |
| Conda software stack | |
| - minimap2 ==2.17 - samtools ==1.12 | |

##### Rule calling\_minimap2\_index

×

Rule properties

|  |  |
| --- | --- |
| Jobs | 1 |
| Input files | |
| - resources/grch38\_with\_alk\_myc\_plasmids.fasta | |
| Output files | |
| - results/calling/index/grch38\_with\_alk\_myc\_plasmids.mmi | |
| Conda software stack | |
| - minimap2 ==2.17 | |

##### Rule calling\_merge\_fastqs

×

Rule properties

|  |  |
| --- | --- |
| Jobs | 2 |
| Input files | |
|  | |
| Output files | |
| - results/calling/merged/{sample,[a-zA-Z\_0-9-]+}\_{read,single|R1|R2}.fastq.gz | |

##### Rule calling\_samtools\_index

×

Rule properties

|  |  |
| --- | --- |
| Jobs | 2 |
| Input files | |
| - results/calling/mapping/{sample}.bam | |
| Output files | |
| - results/calling/mapping/{sample,[a-zA-Z\_0-9-]+}.bam.bai | |
| Conda software stack | |
| - samtools ==1.10 | |

##### Rule calling\_bcf\_index

×

Rule properties

|  |  |
| --- | --- |
| Jobs | 28 |
| Input files | |
| - {prefix}.bcf | |
| Output files | |
| - {prefix}.bcf.csi | |
| Conda software stack | |
| - bcftools =1.11 | |

##### Rule calling\_circle\_coverage\_plot

×

Rule properties

|  |  |
| --- | --- |
| Jobs | 2 |
| Input files | |
| - results/calling/mapping/{sample}.bam - results/calling/mapping/{sample}.bam.bai - results/calling/graphs/{sample}.graph | |
| Output files | |
| - results/calling/coverage\_graphs/{sample,[a-zA-Z\_0-9-]+} | |
| Code | |
| |  |  | | --- | --- | | ``` 1 ``` | ``` cyrcular        graph        plot        {input.bam}        --graph {input.graph}        --output {output.plots}        2> {log} ``` | | |

##### Rule calling\_csv\_report

×

Rule properties

|  |  |
| --- | --- |
| Jobs | 2 |
| Input files | |
| - results/calling/tables/{group}.sorted.annotated.csv - results/calling/coverage\_graphs/{group} | |
| Output files | |
| - results/calling/report/tables/{group} | |
| Code | |
| |  |  | | --- | --- | | ``` 1 2 3 4 ``` | ```         mkdir -p {output}/qc_plots         for f in {input.plots}/*.html; do ( cp "$f" {output}/qc_plots/ ); done         rbt csv-report {input.csv} {output} {params.extra} &> {log} ``` | | |
