# Supplementary material for: Detection and Validation of Circular DNA Fragments Using Nanopore Sequencing
Source: Front Genet. 2022 May 30;13:867018. doi: 10.3389/fgene.2022.867018 (PMC9195511; doi:10.3389/fgene.2022.867018)
Supplement: Supplementary file 2 [file DataSheet1.ZIP › example_report/data/raw/85e24231b9778fcefd9b85abbb3f584123cba2703047e5c2f9fd4d35f7bebfad/prefixes/col_7/13..html]

rbt csv-report


| NUM\_EXONS | page |
| --- | --- |
| 13.0 | 1 |
| 13.0 | 1 |
| 13.0 | 1 |
| 13.0 | 1 |

Back
