# Supplementary material for: Detection and Validation of Circular DNA Fragments Using Nanopore Sequencing
Source: Front Genet. 2022 May 30;13:867018. doi: 10.3389/fgene.2022.867018 (PMC9195511; doi:10.3389/fgene.2022.867018)
Supplement: Supplementary file 2 [file DataSheet1.ZIP › example_report/data/raw/85e24231b9778fcefd9b85abbb3f584123cba2703047e5c2f9fd4d35f7bebfad/prefixes/col_16/MT-.html]

rbt csv-report


| GENES | page |
| --- | --- |
| MT-ATP6;MT-ATP8;MT-CO1;MT-CO2;MT-CO3;MT-CYB;MT-ND1;MT-ND2;MT-ND3;MT-ND4;MT-ND4L;MT-ND5;MT-ND6;MT-RNR1;MT-RNR2;MT-TA;MT-TC;MT-TD;MT-TE;MT-TF;MT-TG;MT-TH;MT-TI;MT-TK;MT-TL1;MT-TL2;MT-TM;MT-TN;MT-TP;MT-TQ;MT-TR;MT-TS1;MT-TS2;MT-TT;MT-TV;MT-TW;MT-TY | 1 |
| MT-ATP6;MT-ATP8;MT-CO1;MT-CO2;MT-CO3;MT-CYB;MT-ND1;MT-ND2;MT-ND3;MT-ND4;MT-ND4L;MT-ND5;MT-ND6;MT-RNR1;MT-RNR2;MT-TA;MT-TC;MT-TD;MT-TE;MT-TF;MT-TG;MT-TH;MT-TI;MT-TK;MT-TL1;MT-TL2;MT-TM;MT-TN;MT-TP;MT-TQ;MT-TR;MT-TS1;MT-TS2;MT-TT;MT-TV;MT-TW;MT-TY | 1 |

Back
