# Supplementary material for: Detection and Validation of Circular DNA Fragments Using Nanopore Sequencing
Source: Front Genet. 2022 May 30;13:867018. doi: 10.3389/fgene.2022.867018 (PMC9195511; doi:10.3389/fgene.2022.867018)
Supplement: Supplementary file 2 [file DataSheet1.ZIP › example_report/data/raw/85e24231b9778fcefd9b85abbb3f584123cba2703047e5c2f9fd4d35f7bebfad/prefixes/col_16/AC0.html]

rbt csv-report


| GENES | page |
| --- | --- |
| AC010145.4;AC010745.1;AC010745.2;AC010745.3;AC010745.4;AC010880.1;AC104623.2;AC113608.1;AC142119.1;CYRIA;ENSG10010134915.1;ENSG10010136800.1;GACAT3;LINC01804;MYCN;MYCNOS;MYCNUT;RN7SL104P;RNU5E-7P;RP11-542H15.1;RP11-549D18.1;RPLP1P5;WI2-2221J1.1 | 1 |
| AC010145.4;AC010745.1;AC010745.2;AC010745.3;AC010745.4;AC010880.1;AC104623.2;AC113608.1;AC142119.1;CYRIA;ENSG10010134915.1;ENSG10010136800.1;GACAT3;LINC01804;MYCN;MYCNOS;MYCNUT;RN7SL104P;RNU5E-7P;RP11-542H15.1;RP11-549D18.1;RPLP1P5;WI2-2221J1.1 | 1 |
| AC010145.4;AC010745.1;AC010745.2;AC010745.3;AC010745.4;AC010880.1;AC104623.2;AC113608.1;AC142119.1;CYRIA;ENSG10010134915.1;ENSG10010136800.1;GACAT3;LINC01804;MYCN;MYCNOS;MYCNUT;RN7SL104P;RNU5E-7P;RP11-542H15.1;RP11-549D18.1;RPLP1P5;WI2-2221J1.1 | 1 |
| AC010145.4;AC010745.1;AC010745.2;AC010745.3;AC010745.4;AC010880.1;AC104623.2;AC113608.1;AC142119.1;CYRIA;ENSG10010134915.1;ENSG10010136800.1;GACAT3;LINC01804;MYCN;MYCNOS;MYCNUT;RN7SL104P;RNU5E-7P;RP11-542H15.1;RP11-549D18.1;RPLP1P5;WI2-2221J1.1 | 1 |

Back
