# Supplementary material for: Detection and Validation of Circular DNA Fragments Using Nanopore Sequencing
Source: Front Genet. 2022 May 30;13:867018. doi: 10.3389/fgene.2022.867018 (PMC9195511; doi:10.3389/fgene.2022.867018)
Supplement: Supplementary file 2 [file DataSheet1.ZIP › example_report/data/raw/8c3f88792c1b6d263d04fc6875e6d123c3bec65f2792ce2056f3131f7f818a3f/indexes/index1.html]

rbt csv-report


rbt report

- 0.38.2
- github

created Fri Jan 28 10:36:06 2022


- csv-report

Loading...

##### EVENT

×

Close

##### Search for EVENT

×

Close

##### CHROM

×

Close

##### Search for CHROM

×

Close

##### start

×

Close

##### Search for start

×

Close

##### ALT

×

Close

##### Search for ALT

×

Close

##### circle\_length

×

Close

##### Search for circle\_length

×

Close

##### num\_segments

×

Close

##### Search for num\_segments

×

Close

##### split\_reads

×

Close

##### Search for split\_reads

×

Close

##### NUM\_EXONS

×

Close

##### Search for NUM\_EXONS

×

Close

##### AF\_nanopore

×

Close

##### Search for AF\_nanopore

×

Close

##### PROB\_PRESENT

×

Close

##### Search for PROB\_PRESENT

×

Close

##### PROB\_ABSENT

×

Close

##### Search for PROB\_ABSENT

×

Close

##### PROB\_ARTIFACT

×

Close

##### Search for PROB\_ARTIFACT

×

Close

##### OTHER\_CHROM

×

Close

##### Search for OTHER\_CHROM

×

Close

##### stop

×

Close

##### Search for stop

×

Close

##### direction

×

Close

##### Search for direction

×

Close

##### length

×

Close

##### Search for length

×

Close

##### GENES

×

Close

##### Search for GENES

×

Close

##### breakpoint\_seq

×

Close

##### Search for breakpoint\_seq

×

Close
