# Supplementary material for: Detection and Validation of Circular DNA Fragments Using Nanopore Sequencing
Source: Front Genet. 2022 May 30;13:867018. doi: 10.3389/fgene.2022.867018 (PMC9195511; doi:10.3389/fgene.2022.867018)
Supplement: Supplementary file 2 [file DataSheet1.ZIP › example_report/data/raw/8c3f88792c1b6d263d04fc6875e6d123c3bec65f2792ce2056f3131f7f818a3f/prefixes/col_3/A[p.html]

rbt csv-report


| ALT | page |
| --- | --- |
| A[pDONR223\_MYC\_WT:4158[ | 1 |
| A[pDONR223\_MYC\_WT:4158[ | 1 |
| A[pDONR223\_MYC\_WT:4158[ | 1 |
| A[pDONR223\_MYC\_WT:4158[ | 1 |
| A[pDONR223\_MYC\_WT:1[ | 1 |
| A[pDONR223\_MYC\_WT:1[ | 1 |
| A[pDONR223\_MYC\_WT:1[ | 1 |
| A[pDONR223\_MYC\_WT:1[ | 1 |
| A[pDONR223\_MYC\_WT:1[ | 1 |

Back
